# *SETD2* mutations in primary central nervous system tumors

**DOI:** 10.1186/s40478-018-0623-0

**Published:** 2018-11-12

**Authors:** Angela N. Viaene, Mariarita Santi, Jason Rosenbaum, Marilyn M. Li, Lea F. Surrey, MacLean P. Nasrallah

**Affiliations:** 1 Department of Pathology and Laboratory Medicine, Children’s Hospital of Philadelphia, University of Pennsylvania Perelman School of Medicine, Philadelphia, PA USA; 2 Department of Pathology and Laboratory Medicine, University of Pennsylvania Perelman School of Medicine, Philadelphia, PA USA; 3 Hospital of the University of Pennsylvania, FO6.089 3400 Spruce St, Philadelphia, PA 19104 USA

**Keywords:** SETD2, Histone, Brain tumor, Glioma, Epigenetics, H3K36me3

## Abstract

Mutations in *SETD2* are found in many tumors, including central nervous system (CNS) tumors. Previous work has shown these mutations occur specifically in high grade gliomas of the cerebral hemispheres in pediatric and young adult patients. We investigated *SETD2* mutations in a cohort of approximately 640 CNS tumors via next generation sequencing; 23 mutations were detected across 19 primary CNS tumors. Mutations were found in a wide variety of tumors and locations at a broad range of allele frequencies. *SETD2* mutations were seen in both low and high grade gliomas as well as non-glial tumors, and occurred in patients greater than 55 years of age, in addition to pediatric and young adult patients. High grade gliomas at first occurrence demonstrated either frameshift/truncating mutations or point mutations at high allele frequencies, whereas recurrent high grade gliomas frequently harbored subclones with point mutations in *SETD2* at lower allele frequencies in the setting of higher mutational burdens. Comparison with the TCGA dataset demonstrated consistent findings. Finally, immunohistochemistry showed decreased staining for H3K36me3 in our cohort of *SETD2* mutant tumors compared to wildtype controls. Our data further describe the spectrum of tumors in which *SETD2* mutations are found and provide a context for interpretation of these mutations in the clinical setting.

## Introduction

Histone modifying enzymes regulate gene expression and play a role in numerous genomic functions through the modification of histones and non-histone proteins [20]. The disruption of normal epigenetic mechanisms secondary to mutations in histone modifying enzymes has been implicated in tumorigenesis [2] and in chemotherapeutic resistance in cancer patients [26]. Specifically, the loss of normal histone modifying enzyme activity is thought to result in alterations in chromatin configuration, disrupting cellular transcription and predisposing a cell to cancerous development [11]. In addition, multiple epigenetic therapies are in development or undergoing testing [23].

The *SETD2* gene encodes SET domain-containing 2 (SETD2), a histone modifying enzyme responsible for all trimethylation of the lysine 36 residue on Histone 3 (H3K36me3) in humans. Decreases in H3K36me3 lead to alterations in gene regulation, increased spontaneous mutation frequency and chromosomal instability [13, 14]. Prior studies have indicated that loss of one allele of *SETD2* does not significantly decrease levels of H3K36me3 [5, 8]; however, it is important to note that biallelic inactivation of *SETD2* may not be the sole mechanism leading to the loss of H3K36me3. For example, overexpression of other proteins such as HOX Transcript Antisense RNA (HOTAIR) can decrease levels of H3K63me3 as well [14].

SETD2-inactivating mutations have been implicated in a number of tumor types (for a review, see [14]). Most frequently, *SETD2* mutations are seen in clear cell renal cell carcinoma (CCRCC) and are thought to confer a poor prognosis [16]. *SETD2* mutations have also been reported in neoplasms of the central nervous system (CNS) [1, 6, 27]. These mutations have been found to be specific to pediatric and young adult high grade gliomas located in the cerebral hemispheres, affecting 15% and 8% of pediatric and adult high grade gliomas, respectively, and not found in other gliomas [6]. In the 2016 WHO Classification of Tumors of the Central Nervous System, *SETD2* mutations are listed under frequent genetic alterations in pediatric (but not adult) high-grade diffuse astrocytic tumors within the cerebral hemispheres [18].

Western blot studies have shown *SETD2*-mutant gliomas have decreased levels of H3K36me3, indicating that the mutations in these tumors are loss-of-function [6]. Similarly, immunohistochemical studies of CCRCC, chondroblastomas, and chordomas have been used to demonstrate decreased staining for H3K36me3 in tumors with *SETD2* mutations [8, 16, 19, 25]. To our knowledge, immunohistochemistry has not been used to evaluate levels of H3K36me3 in *SETD2*-mutant brain tumors.

Here we describe 19 cases of CNS tumors with mutations in *SETD2*. *SETD2* mutation allele frequency and co-occurring mutations in other genes are investigated, and results are correlated with the effects of *SETD2* mutations on epigenetic change, specifically histone methylation and acetylation as shown by immunohistochemistry for H3K36me3, H3K36ac and H3K27me3. Our findings indicate that *SETD2* mutations occur at a wide range of allele frequencies in a variety of tumors of the central nervous system and that those mutations most likely to have functional impact on the gene product are seen most often but not exclusively in pediatric and young adult high grade gliomas of the cerebral hemispheres.

## Materials and methods

This study was approved by an independent institutional review board at the Hospital of the University of Pennsylvania (HUP IRB 827290). All CNS tumors with *SETD2* mutations identified on routine next generation sequencing (NGS) studies performed September 17, 2016 through June 30, 2017 at HUP and from February 1, 2016 to June 30, 2018 at CHOP are included in the current study. Fifteen tumors from HUP and four tumors from Children’s Hospital of Philadelphia (CHOP) are included. Patients whose tumor showed single nucleotide polymorphisms or otherwise benign variants in *SETD2* were excluded.

### Next generation sequencing

At our institutions, targeted NGS of brain tumor specimens is performed as part of routine patient care. Genomic tumor testing was performed at the Center for Personalized Diagnostics (CPD) at the University of Pennsylvania and the Division of Genomic Diagnostics (DGD) at the CHOP, both CLIA-approved laboratories. Genomic DNA from brain tumor specimens is extracted from fresh tissue, formalin-fixed paraffin-embedded tissues or frozen tissue. Tumor DNA is sequenced on an Illumina MiSeq or HiSeq, and the data are analyzed using in-house bioinformatics pipelines.

At the CPD, the Solid Tumor Sequencing panel uses a custom Agilent HaloPlex library preparation (Agilent, Santa Clara, CA) to cover approximately 0.5 megabases, including the entire exonic (coding) sequence of 152 genes, + 10 base pairs of intronic sequence. The 152 genes sequenced on the CPD panel may be found at (https://www.pennmedicine.org/departments-and-centers/center-for-personalized-diagnostics/gene-panels). The library preparation includes unique molecular identifiers to identify duplicate reads. Specimens are sequenced on the Illumina HiSeq 2500 platform (Illumina, San Diego, CA) using multiplexed, paired end reads. Analysis and interpretation is performed using a customized bioinformatics pipeline, Halo_v1.2. All variants are annotated with reference to the hg19 Genome build. Variants are reported according to HGVS nomenclature and classified into 3 categories: Disease-Associated Variants, Variants of Uncertain Significance, and Benign. Variant allele frequency (VAF) is defined as the number of reads of a variant from the reference sequence divided by the total number of reads at that base.

The Comprehensive Solid Tumor Panel v1 at CHOP includes sequence and copy number analyses of 237 cancer genes, and 586 known fusions and many more novel fusions associated with 106 fusion gene partners. The genes included in the panel can be found at (https://www.testmenu.com/chop/Tests/785967). Fusion genes were evaluated by targeted RNA-seq using anchored multiplex PCR with custom designed primers (ArcherDx, Boulder, CO). Full exonic and select intronic/promotor sequence of 237 cancer genes were evaluated by next generation sequencing. Regions of interest were captured using SureSelect^QTX^ target enrichment technology (Agilent Technologies, Santa Clara, CA). Sequencing was performed on Illumina MiSeq or HiSeq (San Diego, CA). Sequencing data were processed using the homebrew software ConcordS v1 and NextGENe v2 NGS Analysis Software (Softgenetics, State College, PA). Variant interpretation was performed according to AMP/ASCO/CAP standards and guidelines for somatic variant interpretation and reporting [15].

### Immunohistochemistry

All tumors with nonsense or frameshift mutations in *SETD2* and those with missense mutations with AF greater than 40% were used for immunohistochemical (IHC) studies. In addition, glioblastomas, anaplastic astrocytomas, pilocytic astrocytomas and meningiomas confirmed to be *SETD2*-wildtype by NGS were used as controls.

H3K36me3 (Abcam ab9050), H3K36ac (Abcam ab177179), and H3K27me3 (Cell Signaling 9733) antibodies were used to stain formalin fixed paraffin embedded slides. Staining was performed on a Bond Max automated staining system (Leica Biosystems). The Bond Refine polymer staining kit (Leica Biosystems) was used. The standard protocol was followed with the exception of the primary antibody incubation which was extended to 1 h at room temperature. The antibodies were used at 1:5 K (H3K36me3), 1:100 (H3K36ac) and 1:150 (H3K36me3) dilutions and antigen retrieval was performed with E1 (H3K36me3 & H3K36ac) or E2 (H3K27me3) (Leica Biosystems) retrieval solution for 20 min. Slides were rinsed, dehydrated through a series of ascending concentrations of ethanol and xylene, then coverslipped.

Immunohistochemistry was scored using the semiquantitative H-score system. H-scores were calculated as 3x the percentage of strongly staining nuclei +2x the percentage of moderately staining nuclei + the percentage of weakly staining nuclei (giving a range of 0 to 300). The H-scores were independently assessed by two neuropathologists board-certified by the American Board of Pathology (MPN and ANV) on de-identified slides.

### TCGA

The Cancer Genome Atlas (TCGA) dataset was retrieved from cbioportal (http://www.cbioportal.org) by searching for *SETD2* mutations in “CNS/Brain” tumors (headings: diffuse glioma, glioblastoma, oligodendroglioma, pilocytic astrocytoma and medulloblastoma). The search was performed 11/30/2017.

### Statistical analysis

Statistical analysis was performed using SPSS version 23.0 (IBM Corp.). Statistical significance was defined as *p* <  .05 and based on two-tailed tests. For the analysis of the number of co-occurring mutations in *SETD2*-mutant tumors, only data from the CPD were used in calculations.

## Results

**Fig. 1:**
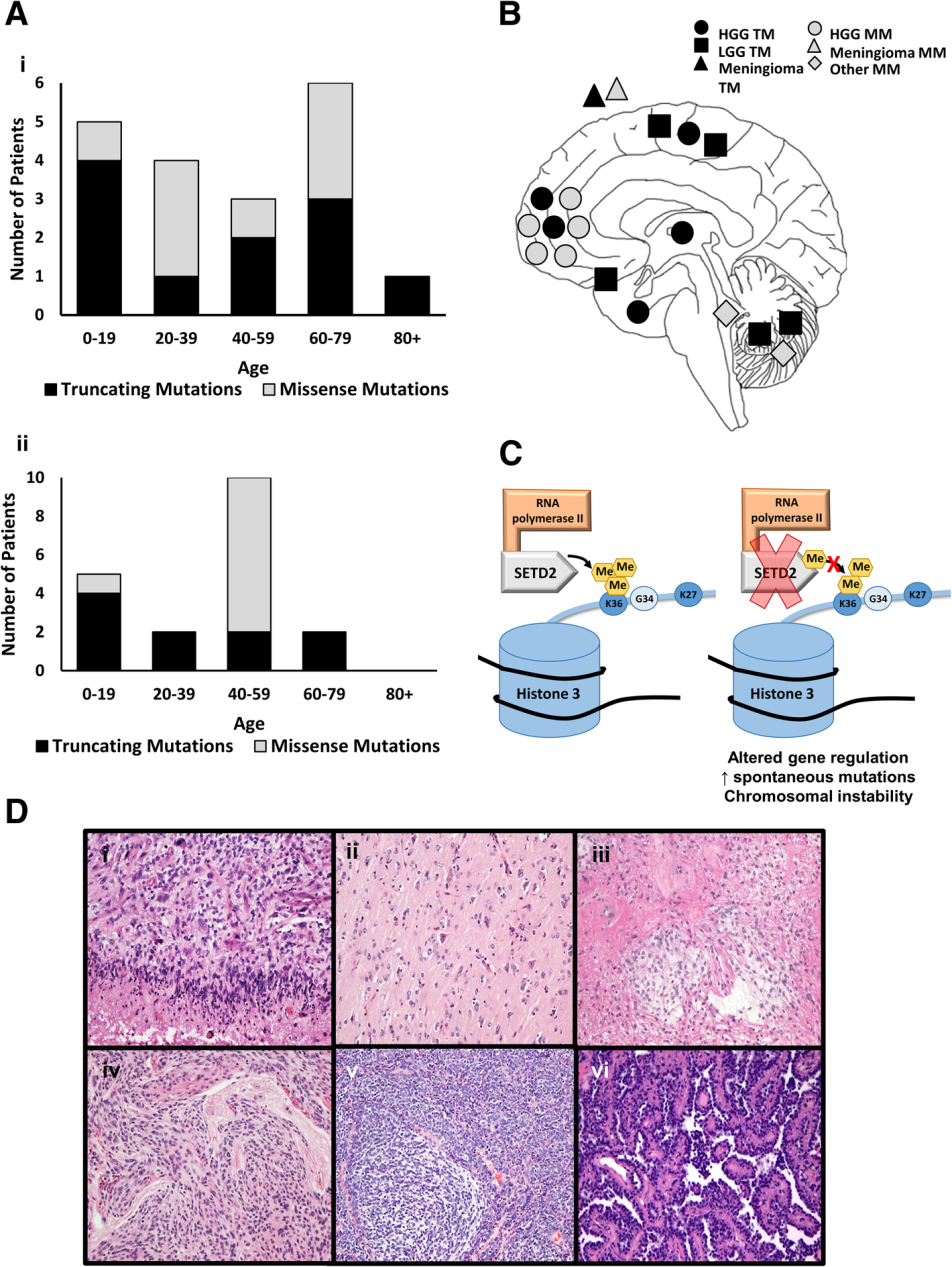
Demographics, locations and histologies of *SETD2* mutant brain tumors. **a** Histograms of patient ages at time of tumor resection for the 19 cases presented in the current study (i) and from the TCGA database (ii). **b** Schematic representation of *SETD2* mutant tumor locations within the CNS. **c** A schematic illustrating the proposed epigenetic effects of *SETD2* alterations. **d** Representative histologies of *SETD2* mutant tumors: Glioblastoma (i), Diffuse astrocytoma (ii), Pilocytic astrocytoma (iii), Atypical meningioma (iv), Medulloblastoma (v), Choroid plexus papilloma (vi). All tumors stained with Hematoxylin and Eosin. All photographs taken at 200x magnification. Truncating mutations (TM), missense mutation (MM)

From September 17, 2016 through June 30, 2017, approximately 400 CNS tumors, including metastases, were sequenced at the University of Pennsylvania CPD. From February 1, 2016 to June 30, 2018 approximately 240 CNS tumors were sequenced at the DGD at CHOP. Nineteen primary brain tumors (fifteen at the University of Pennsylvania and four at CHOP) with *SETD2* mutations were identified on routine NGS studies (Tables 1 and 2). Eleven tumors had nonsense or frameshift mutations (truncating mutations) in *SETD2* and eight had missense mutations. The age of the patients ranged from 9 to 80 years old (mean 43 years, median 42 years), and there was no statistically significant difference (*p* = 0.49) in age between patients with nonsense or frameshift mutations and those with missense mutations (Fig. 1a); however, of high grade gliomas, recurrences often showed missense mutations, whereas frameshift and nonsense mutations were preferentially seen in de novo tumors (Tables 1 and 2). The male to female ratio of the cohort was 3:1.

**Table 1:** Demographics of patients with frameshift and nonsense (truncating) mutations in *SETD2*

Patient #	Age at time of resection	Gender	Location	Diagnosis	Histologic Grade	*SETD2* mutation (AF)	Other disease-associated mutations (AF)^a^	Prior CNS tumor	Follow up from initial tumor resection (months)
1	60	M	Left thalamus	Glioblastoma, IDH-wildtype, WHO grade IV	IV	p.K846lfs*4 (30%)	PTEN p.P246L (47%)	None	8^b^
2	48	F	Left temporal lobe	Glioblastoma, IDH-wildtype, WHO grade IV	IV	p.E282Rfs*9 (4%)	PIK3CA p.G1049R (6%) BRAF p.G466V (2%) NF1 p.F1247Ifs*18 (7%)	None	7^b^
3	37	M	Right frontoparietal lobe	Glioblastoma, IDH-wildtype, WHO grade IV	IV	p.F1132Sfs*22 (23%)	None	None	12^c^
4	55	M	Left frontal lobe	Anaplastic astrocytoma, IDH-wildtype, WHO grade III	III	p.R1598* (44%)	EGFR amplification	None	2^b^
5	75	M	Right frontal lobe	Recurrent/residual glioblastoma, IDH-wildtype	IV	p.W1341* (5%)	ARID1A p.? (3%) FBXW7 p.Q548* (6%) EGFR p.A289V (15%) ARID2 p.Q1215* (11%)	History of glioblastoma resected in 2012 status-post chemoradiation	61^b^
6	80	F	Right frontal lobe resection, gliomatosis cerebri pattern	Diffuse astrocytoma, IDH-wildtype, WHO grade II	II	p.E1907Rfs*4 (6%)	EGFR p.A244T (19%)	None	9^d^
7	10	M	Cerebellum, left hemisphere	Pilocytic astrocytoma, WHO grade I	I	p.R2109* (34%)	KIAA1549-BRAF fusion	None	12^b^
8	16	F	Left temporal lobe	Diffuse astrocytoma	II	p.Q1764Pfs*3 (11%)	QKI-NTRK2 fusion	None	3^b^
9	9	M	Left temporal lobe	Recurrent/residual Pilocytic Astrocytoma	I	p.Q7* (51%)	KIAA1549-BRAF fusion	History of pilocytic astrocytoma resected 2012 (x3) status-post chemotherapy	72^b^
10	17	M	Cerebellum	Pilocytic Astrocytoma	I	p.N261* (28%)	NF1 p.R2269Vfs*11	None	2^b^
11	68	F	Right temporoparietal, extra axial	Atypical meningioma, WHO grade II	II	p.E282Kfs*19 (10%)	NF2 p.L163Wfs*11 (71%)	History of grade I menginomas resected 2005 and 2006	7^a^

Mutation calls were made using transcript ID NM_014159.6

^a^Changes considered variants of uncertain significance are not listed with other disease-associated mutations

^b^No definitive tumor progression detected on surveillance imaging

^c^Surveillance imaging studies not available

^d^Tumor progression suspected on surveillance imaging

**Table 2:** Demographics of patients with missense mutations in *SETD2*

Patient #	Age at time of resection	Gender	Location	Diagnosis	Histologic Grade	*SETD2* point mutation (AF)	Other disease-associated mutations (AF)^a^	Prior CNS tumor	Follow up from initial tumor resection (months)
12	32	M	Right frontal lobe	Recurrent/residual high grade glioma, IDH-mutant	IV	p.G1659D (4%) p.S1268F (3%)	IDH1 p.R132H (49%) CDKN2A p.A36Rfs*17 (72%) NOTCH1 p.? (3%) TP53 p.? (84%) EP300 p.Q2224* (2%)	History of anaplastic astrocytoma resected in 2011 and 2012 status-post resection and chemoradiation	72^c^
13	69	M	Right frontal lobe	Anaplastic astrocytoma, IDH-wildtype, WHO grade III	III	p.I1398T (49%)^b^	EGFR p.G598V (94%) EGFR amplification KMT2C p.S777Kfs*19 (24%)	None	13^d^
14	60	M	Left frontal lobe	Recurrent/residual glioblastoma, IDH-wildtype	IV	p.A2458T (32%) p.S1088F (9%)	MSH6 p.F1088Lfs*5 (9%) TET2 p.W1847* (14%) EZH2 p.? (10%) PTCH1 p.Y1316Tfs*56 (6%) PTEN p.? (4%) TP53 p.H179Y (3%), p.P152L (49%), p.S127F (8%) NF1 p.? (9%), p.W2229* (6%) KDM6A p.? (9%)	History of glioblastoma resected 2013 and 2015 status-post chemoradiation	44^e^
15	64	M	Right frontal lobe	Anaplastic astrocytoma, IDH-wildtype, WHO grade III	III	p.A2242V (49%)^b^	EGFR p.A289T (32%) PTEN p.E7Rfs*17 (18%), p.R130del (7%)	None	2^c^
16	27	F	Right frontal	Recurrent/residual glioblastoma, IDH-mutant	IV	p.E1692K (7%) p.R385K (2%)	IDH1 p.R132H (38%) KMT2C p.? (3%) MEN1 p.? (2%) ARID2 p.Q1604* (2%) BRCA2 p.Q2009* (23%) TP53 p.Y220Pfs*28 (90%)	History of glioblastoma resected 2013 and 2015 status-post chemoradiation	49^f^
17	42	M	Superior saggital sinus, extra axial	Recurrent/residual atypical meningioma	III	p.G1014D (22%)	BRCA2 p.R2494* (3%)	History of atypical meningioma resected 2009 status-post radiation	96^c^
18	33	M	4th ventricle	Choroid plexus papilloma, WHO grade I	I	p.R1089Q (51%)^b^	None	None	5^c^
19	18	M	Cerebellum, left hemisphere	Medulloblastoma, nodular desmoplastic variant, SHH subgroup, WHO grade IV	IV	p.V2371L (5%) p.T1663M (6%)	ATM p.L1327* (42%) CTCF p.R448* (7%) PTCH1 p.C454* (41%) TERT p.? (57%)	None	40^d^

Mutation calls were made using transcript ID NM_014159.6

^a^Changes considered variants of uncertain significance are not listed with other disease-associated mutations

^b^Missense mutations likely represent germline variant

^c^No definitive tumor progression detected on surveillance imaging

^d^Patient had tumor recurrences and resections, now with no definitive progression detected on surveillance imaging

^e^Surveillance imaging studies not available

^f^Tumor progression suspected on surveillance imaging

Twelve of these *SETD2*-mutant tumors were located within the cerebral hemispheres while seven occurred outside the hemispheres (two extra-axial, one thalamic, and four posterior fossa; Fig. 1b). A broad range of tumor histologies were seen, including high grade gliomas (*n* = 10, 62.5%, with 4 of them recurrent), low grade astrocytic tumors (*n* = 5, 12.5%), atypical meningiomas (*n* = 2, 12.5%), a medulloblastoma (*n* = 1, 6.3%) and a choroid plexus papilloma (*n* = 1, 6.3%). Examples of tumor histology are show in Fig. 1d. Overall, eleven of the *SETD2*-mutant tumors were classified as high grade (WHO grade III or IV) and eight were low grade tumors (WHO grade I or II) (Tables 1 and 2).

In total, 23 *SETD2* changes amongst the 19 tumors were detected at a wide range of VAF (range 2–51%); 4 tumors had more than one *SETD2* missense mutation, 3 of which were recurrent high grade gliomas, and the fourth a medulloblastoma. No statistically significant difference (*p* = 0.49) in VAF was seen between truncating mutations and missense mutations.

**Fig. 2:**
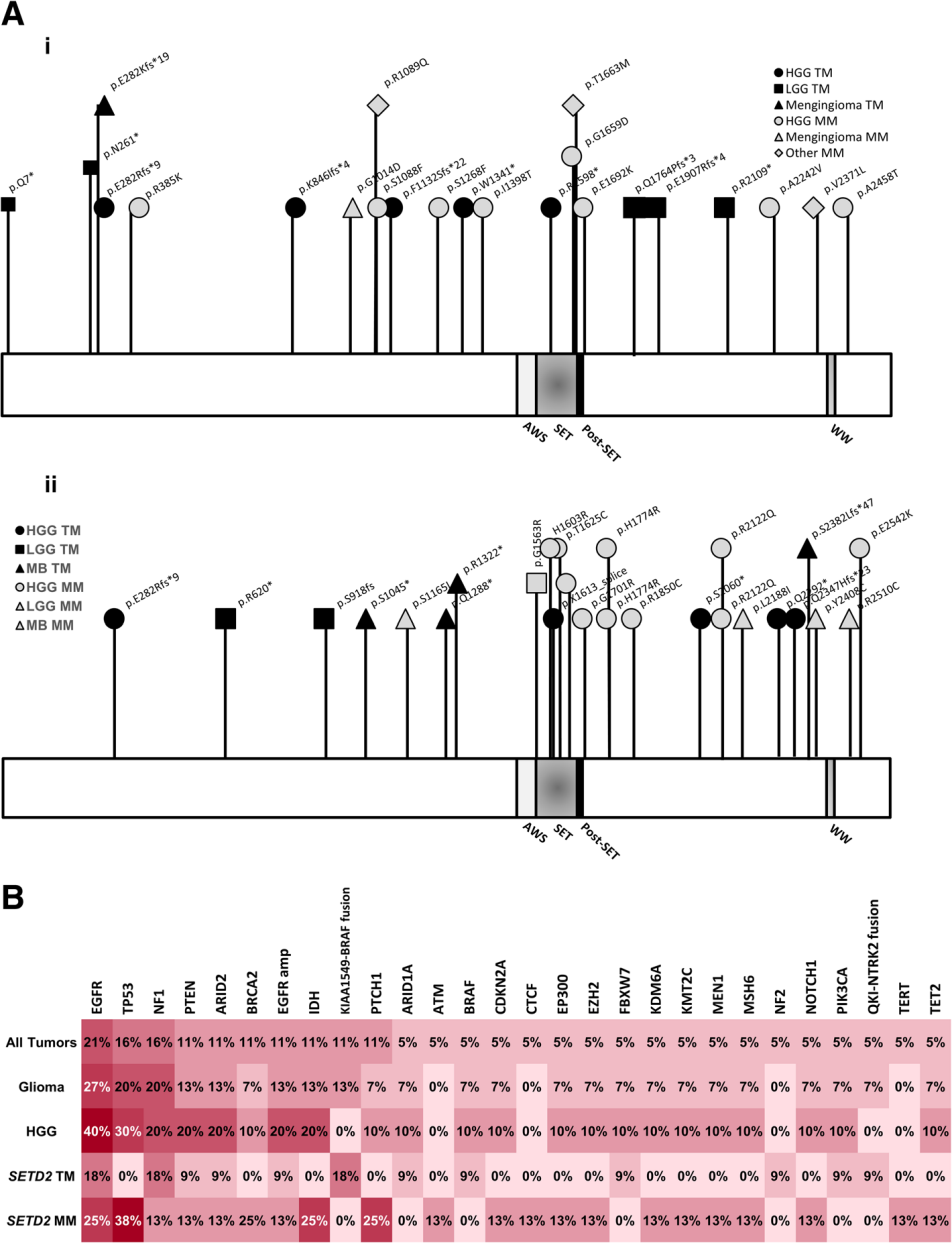
Location of mutations within *SETD2* and co-occurring pathogenic mutations. **a** Schematic representation of the locations of mutations in SETD2 for the 16 cases presented in the current study (i) and the TCGA database (ii) (AWS, Associated with SET). **b** Other pathogenic mutations co-occurring in tumors with *SETD2* mutations with the percentage of tumors with each mutation is labeled within each cell. Darker shading corresponds to higher percentages. Truncating mutations (TM), missense mutation (MM)

The detected mutations were distributed throughout *SETD2* with the majority of the high grade glioma nonsense or frameshift mutations occurring 5′ to the SET domain (VAF 4–44%) (Fig. 2a). The nonsense or frameshift mutations for the low grade gliomas occurred throughout the SETD2 gene (VAF 6–34%).

Missense mutations occurred throughout the *SETD2* gene, and in gliomas, were found predominantly in recurrent high grade gliomas, including recurrent glioblastomas (Table 2). The exceptions were patients 10 and 12. For patient 10 in Table 2, diagnosed with an *IDH*-wildtype anaplastic astrocytoma, a p.I1398T change in SETD2 was found; however, this may represent a benign single nucleotide polymorphism as it is seen at > 0.1% frequency in the Ashkenazi Jewish population (http://gnomad.broadinstitute.org/) [12]. Patient 12, with otherwise similar characteristics, showed *SETD2* p.A2242V, which we classify as a variant of uncertain significance, given that is not been identified previously. The remaining missense mutations found in *SETD2* may represent changes found with the increased mutational load seen with tumor recurrence in this cohort (4.57 ± 3.40 mutations in recurrent tumors vs. 1.41 ± 1.24 mutations in primary tumors, *p* < 0.01), and known in the literature, particularly after treatment with temozolomide [3, 9]. The largest number of co-occurring mutations (11) was seen in patient 14 following chemotherapy and radiation. Of note, this patient also had two MM in *SETD2*. Within the adult cohort, tumors with missense mutations in *SETD2* had more concurrent mutations than did those with truncations of *SETD2* (5.17 ± 3.31 vs. 1.50 ± 1.35, *p* < 0.05). Mutations in *EGFR* were found to be the most commonly co-occurring change with *SETD2* changes and were seen in 40% of the high grade gliomas in this cohort, similar to the frequency of EGFR mutations found in high grade gliomas overall (Fig. 2b) [1]. Pathogenic mutations in *TP53* were seen in 30% of high grade gliomas with *SETD2* changes, and IDH mutations were seen in 20%. However, *TP53* and *IDH* mutations were only seen in tumors with missense mutations in *SETD2* and not those with nonsense or frameshift mutations in *SETD2*. No mutations in *H3F3A* were seen to co-occur with *SETD2* changes.

### Immunohistochemistry

**Fig. 3:**
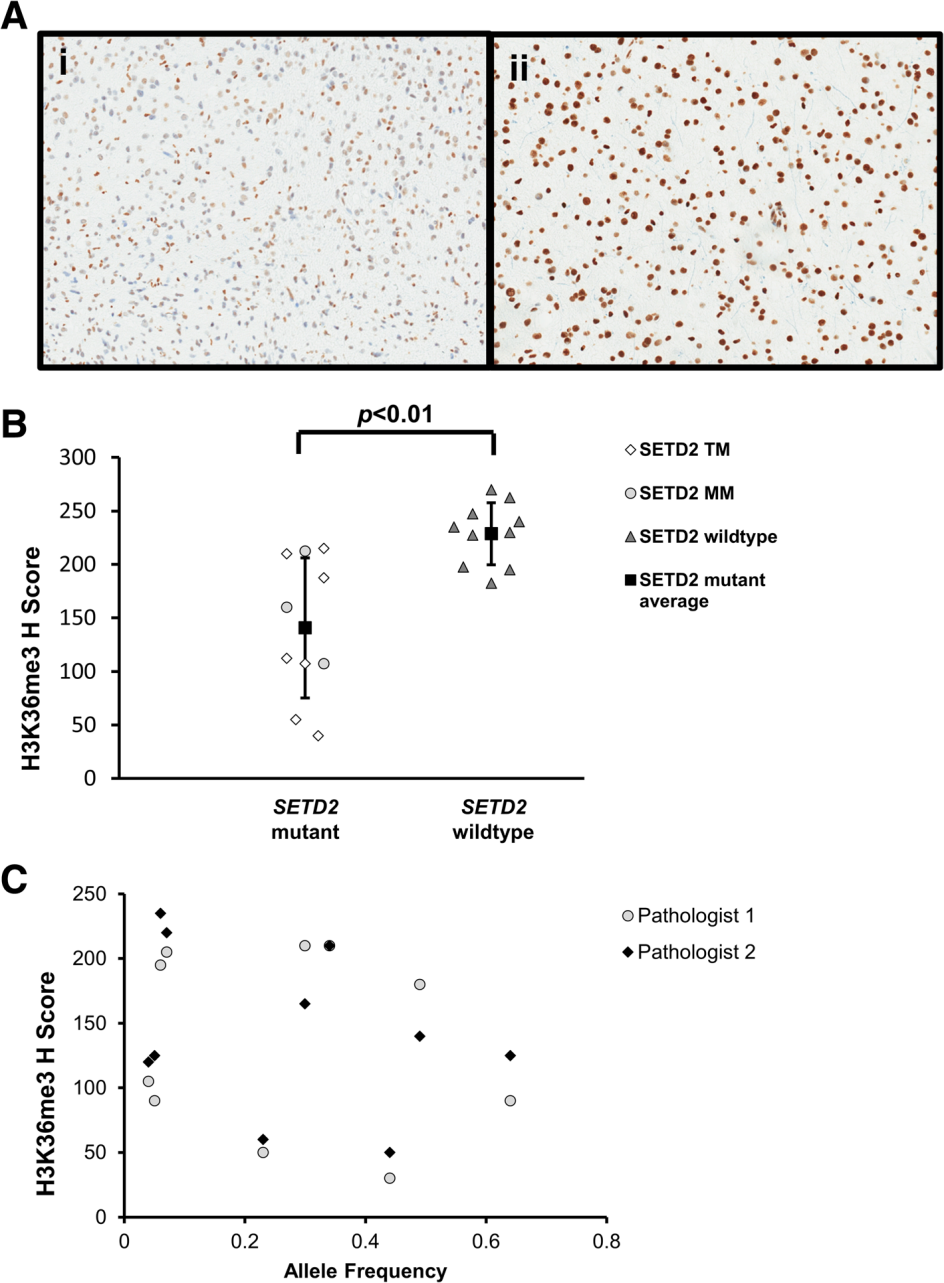
Immunohistochemical staining for H3K36me3. **a** Examples of immunohistochemical staining for H3K36me3 in high grade gliomas with a truncating mutation in *SETD2* (i) and wildtype control (ii); both images were taken at 200x magnification. **b** H scores for *SETD2* mutant tumors and wildtype tumors. Averages for each group are shown as black squares and with error bars representing the standard deviation. **c** H scores calculated by two independent pathologists for *SETD2* mutants plotted against allele frequency. Truncating mutations (TM), missense mutation (MM)

Immunohistochemistry for H3K36me3, H3K36ac and H3K27me3 expression were assessed with H-scores by two neuropathologists (ANV and MPN) on *SETD2*-mutant tumors and histologic controls confirmed to be wildtype for *SETD2* by NGS. Gliomas with SETD2 mutations showed significantly lower H-scores for H3K36me3 compared to wildtype controls (140.8 ± 65.4 vs. 228.8 ± 29.1, *p* < 0.01) (Fig. 3). In contrast, statistically significant differences in staining for H3K36ac and H3K27me3 between SETD2 mutants and controls were not observed (H3K36ac: 140.1 ± 35.7 vs. 160.0 ± 41.8, *p* = 0.13; H3K27me3: 206.1 ± 24.2vs. 190.8 ± 33.5, *p* = 0.23). There was no correlation between AF and H-score for any of the immunohistochemical markers [H3K36me3: *F*_1,8_ = 0.55, *p =* .48 with an R^2^ of 0.06 (Fig. 3c); H3K36ac: *F*_1,8_ = 0.54, *p =* .48 with an R^2^ of 0.06; H3K27me3: *F*_1,8_ = 0.46, *p =* .53 with an R^2^ of 0.08]. The concordance correlation coefficients for the three antibodies were as follows: H3K36me3 concordance correlation coefficient of 0.88 [95% CI 0.69–0.96]; H3K36ac concordance correlation coefficient of 0.58 (95% CI 0.12–0.83); H3K27me3: concordance correlation coefficient of 0.51 (95% CI 0.16–0.75).

### TCGA

**Fig. 4:**
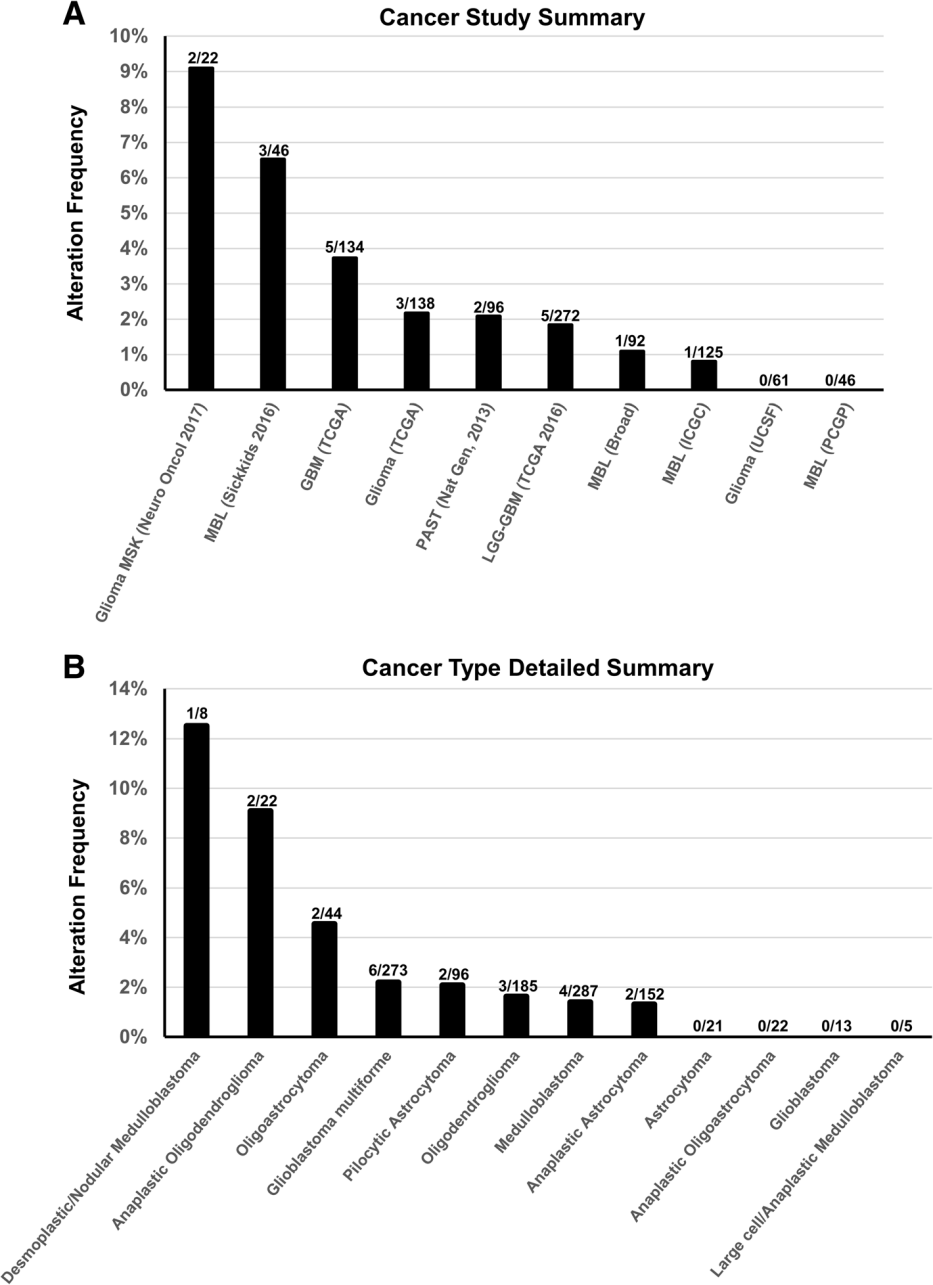
*SETD2* mutations in CNS tumors retrieved from the TCGA database. **a** Frequency of *SETD2* alterations detected per study. **b** Frequency of *SETD2* alterations detected per tumor type. The number of cases with SETD2 variants over the denominator of the total number of analyzed cases for each group is indicated above the bars

The results shown here are based upon data generated by the TCGA Research Network: http://cancergenome.nih.gov/, via http://www.cbioportal.org/ [4, 7]. A total of 22 CNS tumors with *SETD2* mutations were identified across the cohorts included in the TCGA datasets, with 0–14% of CNS tumors harboring a *SETD2* mutation depending on the cohort (Fig. 4). Eleven tumors had truncating mutations, ten had missense mutations and one had a splice-site mutation. The age of the patients ranged from 2 to 74 years (mean 40.1 ± 23.6 years) with a male to female ratio of 2.3:1. There was no statistically significant difference (*p* = 0.22) in age between patients with truncating mutations and those with missense mutations. *SETD2* mutations were found in high grade gliomas (*n* = 14, 63%), low grade gliomas (*n* = 3, 14%), and medulloblastomas (*n* = 5, 23%). The low grade gliomas included two pilocytic astrocytomas and an IDH-mutant, 1p/19q-codeleted, WHO grade II oligodendroglioma. Data on the location of the tumors is limited; a study of glioblastomas found that all tumors with *SETD2* mutations were located in the cerebral hemispheres [6]. However, it is likely *SETD2* mutant tumors were also present in the posterior fossa as mutations were seen in 5 medulloblastomas and 2 pilocytic astrocytomas, tumors which both have a strong association with the posterior fossa. In total, 26 *SETD2* mutations were seen among the 22 tumors with one medulloblastoma having 3 *SETD2* missense mutations. For the 11 tumors for which data on AF was available, the frequency ranged from 5 to 48%. No statistically significant difference in AF was seen between truncating mutations and missense mutations (*p* = 0.82). The mutations were distributed throughout SETD2 (Fig. 2 aii). Survival data is available on 16 patients (15 patients with gliomas and 1 patient with medulloblastoma). The average follow-up was 19.1 ± 17.3 months (range 5 to 72 months) for all tumors and 15.8 ± 11.4 months (range 5 to 45 months) for patients with high grade gliomas. Twelve patients were still living. High grade gliomas with TM had an average follow-up of 13.2 ± 10.8 months and those with MM had an average follow-up of 17.7 ± 12.3 months (*p* = .52). Four deceased patients all had high grade gliomas (two of which were recurrent) with an average survival of 16.3 ± 10.0 months (range 7 to 30 months). Two of the deceased patients had TM (survival 7 months and 30 months) and two patients had MM (survivals of 11 and 17 months).

## Discussion

Epigenetic changes, such as DNA methylation and histone modifications affect the structure of chromatin, and therefore have a potentially broad impact on transcription. Epigenetic changes have been detected in a wide range of tumors, and a variety of drugs have been developed to target these changes; for a review, see [23]. Epigenetic mutations in gliomas include H3 K27 M mutations in diffuse midline gliomas and H3.3 G34R/V mutations in gliomas of the cerebral hemispheres [18]. In diffuse midline gliomas, the H3 K27 M mutation has been shown to globally reduce levels of H3K27me3 levels, altering transcription and driving tumorigenesis. Several therapies that target enzymes responsible for chromatin modifications in these gliomas have been developed; for a review, see [17]. In gliomas, mutations have also been reported in *SETD2*, whose protein product is an enzyme responsible for trimethylation of the lysine 36 residue on Histone 3 (H3K36me3) in humans. These mutations potentially decrease H3K36me3, which may alter gene regulation, increase spontaneous mutation frequency and lead to chromosomal instability, with theoretically targetable effects (Fig. 1c).

Our investigation of *SETD2* mutations in brain tumors yields findings consistent with the previous report of *SETD2* mutations in gliomas, which shows that loss-of-function *SETD2* mutations occur in older children and young adults in high grade gliomas of the cerebral cortex [6]. In addition, we expand on those results to demonstrate that subclonal mutations in *SETD2* are seen in a broad range of tumors and locations. Prior studies focusing on gliomas illustrate that *SETD2* mutations are found nearly exclusively within the cerebral hemispheres [1, 6, 22, 27]. Similarly, in our cohort, mutations were most commonly seen in high grade gliomas within the cerebral hemispheres (12 of 19 tumors). However, our results include a broader range of CNS tumor types, and demonstrate *SETD2* truncating mutations in atypical meningiomas and pilocytic astrocytomas, as well as missense mutations in a choroid plexus papilloma and a medulloblastoma. Data from the TCGA database also showed *SETD2* mutations in pilocytic astrocytomas, an oligodendroglioma, and medulloblastomas. Prior studies of medulloblastomas did not detect mutations in *SETD2* [10] suggesting *SETD2* mutations are rare in these tumors. The medulloblastoma described in our cohort belongs to the Sonic Hedgehog genetic group, and has two point mutations in *SETD2* with reported VAFs of 5% and 6%.

In contrast to previous work showing that *SETD2* mutations are seen in high grade gliomas but not low grade gliomas, we found frameshift mutations in *SETD2* in two diffuse astrocytomas, WHO grade II. However, one *SETD2*-mutant astrocytoma in an older patient (#6) also harbors an *EGFR* mutation and lacks *IDH* changes, and so is in essence a “molecular glioblastoma,” in addition to radiologically showing a *gliomatosis cerebri* pattern of growth. Given the previous findings that *SETD2* mutations are specific to high grade gliomas, the *SETD2* change in this tumor may be hypothesized to indicate or correlate with aggressive behavior. The patient experienced slow radiological progression and clinical decline over 8 months despite temozolomide therapy, and subsequently transitioned to hospice and comfort care. Along the same lines, we found truncating mutations in three pilocytic astrocytomas. Although pilocytic astrocytomas are grade I tumors, they can show anaplastic changes, and one of these three tumors did have increased mitotic activity (#7). Additionally, another pilocytic astrocytoma was a recurrent tumor with a history of three prior resections and chemotherapy (#9).

In addition, not all patients conform to the previously reported age and tumor location profile. For example, the patient diagnosed with the diffuse astrocytoma mentioned above was 80 years old at the time of diagnosis. Also, a 60-year-old patient presented with a thalamic glioblastoma, which demonstrated a frameshift mutation in *SETD2* at a 30% VAF. Data from the TCGA database also showed a wide range of patient ages (2–74 years).

In our cohort, mutations were seen in a variety of regions within the *SETD2* gene and at a broad range of VAF in tumors. In high grade gliomas, nonsense and frameshift mutations were mostly located 5′ to the SET domain. These findings are similar to what has been reported [6]. In contrast, in the low grade astrocytic tumors, nonsense or frameshift mutations often occurred 3′ to the SET domain, including in tumor #6. Missense mutations were found throughout *SETD2*. The significance of the location of mutation with respect to nonsense mediated decay of the RNA is unknown. *SETD2* mutations with low VAF (defined as VAF < 10%), were seen to co-occur with an average of 3.8 ± 1.7 other mutations (range 1–6 mutations). Those tumors with higher *SETD2* mutation VAF (≥10%) had an average of 1.8 ± 2.9 additional co-occurring mutations (range 0–11 mutations).

Several tumors in our cohort were recurrent/residual gliomas. Sequencing for *SETD2* mutations was not performed on the prior resection specimens. However, one patient (#13) had tumor recurrence and a subsequent resection which showed the same *SETD2* mutation (p.I1398T) at a similar VAF. Patients 13, 15, and 18 had MM occurring at VAF around 50%. It is possible that these mutations are germline though this cannot be confirmed as paired normal sequencing for *SETD2* was not performed.

We attempted to determine whether the *SETD2* mutation resulted in a functional effect through immunohistochemical studies of epigenetic markers. If *SETD2* mutations are indeed driving tumorigenesis in some CNS tumors, the exact mechanism by which this occurs also requires further elucidation. One of the leading hypotheses suggests that loss of *SETD2* function in tumor cells decreases levels of H3K36me3, which subsequently leads to alterations in gene regulation, increased spontaneous mutation frequency and chromosomal instability (Fig. 1c) [13, 14]. Evidence also indicates that increased levels of H3K36ac are seen when the levels of H3K36me3 decrease [21]. We employed IHC for H3K36me3, H3K36ac and H3K27me3 to investigate the impact of *SETD2* mutations on histone methylation and acetylation. We hypothesized that decreased H3K36me3 staining and increased staining for H3K36ac would be present in *SETD2* mutant tumors with mutation seen at high VAF. Additionally, prior investigations have shown that cells depleted for all H3K36-directed methyltransferases have a reduction in H3K36me3 and also have elevated levels of H3K27me3 [19]. Based on these findings, we investigated whether staining for H3K27me3 is increased in *SETD2* mutant tumors.

Immunohistochemical staining for H3K36me3 showed a statistically significant decrease in staining for *SETD2* mutant gliomas compared to *SETD2* wildtype histologic controls. However, given the variability in staining and the lack of correlation with allele frequency, IHC for the detection of *SETD2* mutations is impractical in a clinical setting. Prior work in gliomas also found a significant decrease in levels of H3K36me3 in gliomas with heterozygous mutations in *SETD2* by Western Blot [6]. In contrast, studies have indicated that bi-allelic loss of *SETD2* is needed to significantly decrease levels of H3K36me3 in in vitro models and renal cell carcinoma [5, 8, 14]. For example, the most significant decreases in staining for H3K36me3 in non-CNS tumors with *SETD2* mutations were seen when both *SETD2* allelic copies were lost [8, 16, 19, 25]. Specifically, studies of clear cell renal cell carcinoma indicate that mutations occurring at higher AFs may not result in significant decreases in H3K36me3 unless both *SETD2* alleles are affected [5, 8]. Further studies are needed to assess whether this statistically significant decrease in levels of H3K36me3 in gliomas with heterozygous mutations in *SETD2* has a function impact on tumorigenesis, as well as to determine if there is loss of the alternate allele.

Immunohistochemical stains for H3K36ac and H3K27me3 did not show statistically significant differences in staining between mutants and controls, although an increase in staining for H3K27me3 were seen in *SETD2* mutants compared to controls. There was no correlation between VAF and levels of staining for any antibodies though this may be due to the small number of tumors evaluated.

An alternative mechanistic hypothesis is that *SETD2* mutations are interacting with other mutations to drive tumorigenesis. For example, SETD2 may bind to p53 and regulate the transcription of specific genes [28]. Studies of lung adenocarcinomas found loss of H3K36me3 lead to accelerated progression of early- and late-stage tumors; however SETD2 loss alone was not sufficient to overcome the p53-regulated barrier that suppresses the formation of higher grade adenocarcinomas [24]. It is possible that *SETD2* mutations work in conjunction with other mutations such as *TP53* or mutations in growth factor pathways to promote tumorigenesis. Although one of the most frequently observed concurrently mutated genes in our cohort of high grade gliomas is *TP53*, *TP53* mutations were only seen in tumors with *SETD2* missense mutations and not in those tumors with *SETD2* nonsense or frameshift mutations. Most often, the co-occurrence of *SETD2* and *TP53* mutations was seen in recurrent gliomas, and the VAFs varied. These findings do not lend support to the hypothesis that *SETD2* mutations synergize with *TP53* mutations, and further studies are necessary.

In addition to *TP53*, the other most frequently observed genes showing mutations concurrent with *SETD2* mutation within the high grade glioma subset were *EGFR* and *PTEN*, likely due to the frequency of mutation in these genes in glioblastoma. Recurrent tumors with *SETD2* mutations had significantly more concurrent mutations than did first occurrence *SETD2*-mutant tumors. *IDH* mutations were present in a subset (18%) of diffuse gliomas with *SETD2* changes, which in this study were all *SETD2* missense mutations rather than nonsense or frameshift mutations. Concurrent mutations in *SETD2* and *H3F3A* were not seen. Both of these findings are consistent with prior investigation [6].

Data on the long-term survival for patients with *SETD2*-mutant CNS tumors is limited. Twelve of sixteen patients from the TCGA cohort were still living, and the data on follow-up does not allow any conclusions to be drawn regarding the impact of the SETD2 mutations. At the conclusion of the current study, all patients were still living. Focusing on patients with high grade gliomas, the average follow-up period from initial presentation was 18.0 ± 24.3 months (range 2 to 61 months) for truncating mutations and 36.0 ± 28.3 months (range 2 to 72 months) for missense mutations. Two tumors with *SETD2* missense mutations were positive for *IDH1* mutations; when comparing tumors with *SETD2* missense mutations, *IDH*-mutant tumors had follow-up periods of 79 and 42 months versus follow-up periods ranging from 2 to 44 months with an average follow-up of 19.7 ± 21.8 months for *IDH*-wildtype gliomas. On average, longer follow-up data was available for patients with tumors with missense mutations as several of these were identified in recurrences (4 of 5 high grade gliomas with missense mutations had recurrences available for analysis). It is important to note that the initial tumor resections of three of these tumors were not sequenced for *SETD2*, so it is uncertain if the changes were present at the time of initial presentation. However, one patient (#13) had an *IDH*-wildtype anaplastic astrocytoma recur with progression to glioblastoma; the initial frontal lobe resection and the frontal lobe recurrence demonstrated the same mutational profile, including the same *SETD2* missense mutation, whereas an intervening temporoparietal tumor resection demonstrated different mutations. EGFR p.G589 V and a copy number gain of EGFR were detected in both frontal lobe resections, and EGFR p.T263P and PTEN p.T366Hfs*50 were detected in the temporoparietal resection. In these recurrent/residual high grade gliomas with changes in *SETD2*, longer durations of follow-up were seen after initial presentation (mean of 79 and 42 months for *IDH*-mutant tumors and 52.5 ± 12.0 months for *IDH*-wildtype tumors) than is common for high grade gliomas. Further studies are required to better elucidate how nonsense or frameshift mutations and missense mutations in *SETD2* relate to prognosis.

Another question that requires further investigation is the difference between truncating mutations and missense *SETD2* mutations. Prior Western blot studies found a decrease in levels of H3K36me3 for both truncating and missense mutations [6]. As a number of different missense mutations occurring throughout the *SETD2* gene have been seen in CNS tumors, as well as in other tumor types, the role of individual missense mutations in tumorigenesis is unclear. One possibility is that a subset of missense mutations are passenger mutations that occur following glioma therapy. Three recurrent high grade gliomas (#12, 14 and 16) in our subset had multiple missense mutations in *SETD2*. In these cases, a higher mutational burden was present following chemoradiation. It is likely that the missense *SETD2* mutations seen at low VAF are not drivers of tumorigenesis in these tumors. In contrast, for the tumors with nonsense or frameshift mutations in *SETD2*, fewer concurrent PM were detected, and in one tumor (#3), the PM in *SETD2* was the only mutation detected on the NGS panel, at a VAF of 23%.

## Conclusions

In summary, these findings suggest that *SETD2* mutations, although most common in high grade gliomas of the cerebral hemispheres, may be found in a variety of primary CNS tumors and locations. Immunohistochemistry shows a decrease in H3K36me3 in tumor with *SETD2* mutations, implicating epigenetic pathways in tumor biology. Additional studies are needed to investigate the role of *SETD2* mutations in tumorigenesis.

## Abbreviations

CCRCC: Clear cell renal cell carcinoma
CHOP: Children’s Hospital of Philadelphia
CNS: Central nervous system
CPD: Center for Personalized Diagnostics
DGD: Division of Genomic Diagnostics
H3K27me3: Trimethylation of the lysine 27 residue on Histone 3
H3K36ac: Acetylation of the lysine 36 residue on Histone 3
H3K36me3: Trimethylation of the lysine 36 residue on Histone 3
HOTAIR: HOX Transcript Antisense RNA
HUP: Hospital of the University of Pennsylvania
IHC: Immunohistochemistry
MM: Missense mutation
NGS: Next generation sequencing
SETD2: SET domain-containing 2
TCGA: The Cancer Genome Atlas
TM: Truncating mutation
VAF: Variant allele frequency
